# Health facility and skilled birth deliveries among poor women with *Jamkesmas* health insurance in Indonesia: a mixed-methods study

**DOI:** 10.1186/s12913-017-2028-3

**Published:** 2017-02-02

**Authors:** Mohamad I. Brooks, Hasbullah Thabrany, Matthew P. Fox, Veronika J. Wirtz, Frank G. Feeley, Lora L. Sabin

**Affiliations:** 1 Pathfinder International, 9 Galen St, Suite 217, Watertown, 02472 MA USA; 2 Department of Global Health, Boston University School of Public Health, 801 Massachusetts Avenue, Crosstown 3rd Fl, Boston, 02118 MA USA; 3 Center for Health Economics and Policy Studies, University of Indonesia School of Public Health, Building G Room 311, Depok, 16424 West Java Indonesia; 4 Department of Epidemiology, Boston University School of Public Health, 715 Albany Street, Talbot T2C, Boston, 02118 MA USA; 5 Center for Global Health and Development, Boston University School of Public Health, 801 Massachusetts Avenue, Crosstown 3rd Fl, Boston, 02118 MA USA

**Keywords:** Health insurance, Universal health coverage, Jamkesmas, Indonesia, Poor, Maternal health, Health facility delivery, Institutional delivery, Skilled birth delivery, Skilled birth attendant

## Abstract

**Background:**

The growing momentum for quality and affordable health care for all has given rise to the recent global universal health coverage (UHC) movement. As part of Indonesia’s strategy to achieve the goal of UHC, large investments have been made to increase health access for the poor, resulting in the implementation of various health insurance schemes targeted towards the poor and near-poor, including the *Jamkesmas* program. In the backdrop of Indonesia’s aspiration to reach UHC is the high rate of maternal mortality that disproportionally affects poor women. The objective of this study was to evaluate the association of health facility and skilled birth deliveries among poor women with and without *Jamkesmas* and explore perceived barriers to health insurance membership and maternal health service utilization.

**Methods:**

We used a mixed-methods design. Utilizing data from the 2012 Indonesian Demographic and Health Survey (*n* = 45,607), secondary analysis using propensity score matching was performed on key outcomes of interest: health facility delivery (HFD) and skilled birth delivery (SBD). In-depth interviews (*n* = 51) were conducted in the provinces of Jakarta and Banten among poor women, midwives, and government representatives. Thematic framework analysis was performed on qualitative data to explore perceived barriers.

**Results:**

In 2012, 63.0% of women did not have health insurance; 19.1% had *Jamkesmas*. Poor women with *Jamkesmas* were 19% (OR = 1.19 [1.03–1.37]) more likely to have HFD and 17% (OR = 1.17 [1.01–1.35]) more likely to have SBD compared to poor women without insurance. Qualitative interviews highlighted key issues, including: lack of proper documentation for health insurance registration; the preference of pregnant women to deliver in their parents’ village; the use of traditional birth attendants; distance to health facilities; shortage of qualified health providers; overcrowded health facilities; and lack of health facility accreditation.

**Conclusions:**

Poor women with *Jamkesmas* membership had a modest increase in HFD and SBD. These findings are consistent with economic theory that health insurance coverage can reduce financial barriers to care and increase service uptake. However, factors such as socio-cultural beliefs, accessibility, and quality of care are important elements that need to be addressed as part of the national UHC agenda to improve maternal health services in Indonesia.

## Background

The growing momentum for quality and affordable health care for all has given rise to the recent global universal health coverage (UHC) movement [33]. As part of Indonesia’s strategy to achieve the goal of UHC, considerable investments have been made to increase health access for the poor [23, 36]. This strategy is in line with WHO recommendations to improve equity by reducing out-of-pocket expenditures for poor households and allocating more resources to those in need [34]. The result of Indonesia’s UHC initiative is the roll-out and implementation of various health insurance schemes targeted towards the poor and near-poor, including the *Jaminan Kesehatan Masyarakat* (*Jamkesmas*) [health insurance for the population] program.

In 2004, the *Asuransi Kesehatan Masyarakat Miskin* (*Askeskin*) program in Indonesia established health insurance coverage for the poor [23, 36]. The *Askeskin* program was designed to increase access and quality of health services for the poor through operational funds provided to *puskesmas* [community health center] in the form of capitation payments and a fee-for-service health insurance scheme reimbursed through a quasi-governmental agency. The *Askeskin* program provided block grants through this agency to target the poor through the distribution of *Askeskin* health insurance cards and payment of hospital claims. In 2008, the *Askeskin* program was expanded to include the near-poor as part of the new *Jamkesmas* program. *Jamkesmas* beneficiaries were required to sign up for health insurance cards. Households in the lowest two wealth quintiles were eligible for *Jamkesmas* and targeting was done through a mixture of geographic and proxy means testing. The benefits of *Jamkesmas* included free health services in community health centers and 3rd class wards (basic level) in government hospitals and designated private hospitals. *Jamkesmas* beneficiaries were entitled to comprehensive maternity benefits, including antenatal care, institutional delivery, and postnatal care [23, 36].

Even though total health expenditures (THE) per capita increased from US$20 to US$107 between 2002 and 2012, Indonesia’s expenditure on health as measured by the percent of gross domestic product (GDP) on total health expenditure is low compared to the other countries in the WHO South-East Asia Region (SEAR): 2.9% compared to regional average of 4.2% in 2012 [37]. The low proportion of GDP invested in health is one factor contributing to the high rate of maternal mortality in Indonesia.

Although Indonesia has reduced its maternal mortality rate (MMR) over the last 25 years, this decrease has been progressing at a much slower rate compared to the SEAR average. With an MMR of 126 deaths per 100,000 live births in 2015 [37], Indonesia did not meet Millennium Development Goal (MDG) 5 to reduce MMR to 102 deaths per 100,000 live births by 2015. One of the main factors contributing to Indonesia’s high rate of maternal mortality is the lack of access to maternal health services, especially among the poor. For example, 88.1% of women in the highest wealth quintile delivered in a health facility compared to 29.7% of women in the lowest wealth quintile in 2012 [5].

A recent World Bank report provides evidence of the positive impact that UHC in Low and Middle Income Countries (LMIC) can have regarding access to health care; however, the evidence on financial protection is limited and even less convincing for improving health outcomes [3]. In regards to improvements in maternal health, studies from Mexico, Peru, Argentina and Bangladesh have shown that UHC interventions can result in increased antenatal visits, health facility deliveries, and postnatal care visits [8, 9, 17, 28].

In order to better understand the effects of UHC interventions on the use of maternal health services in Indonesia, we evaluated the potential role of the *Jamkesmas* health insurance scheme in increasing health facility and skilled birth delivery among poor women in Indonesia, and explored the reasons behind any observed changes.

## Methods

We used an explanatory mixed-methods design [7]. For the quantitative component, secondary analysis of the nationally representative Indonesian Demographic and Health Survey (IDHS) from 2012 (*n* = 45,607) [5] was used to assess the proportion of women with health insurance coverage and to measure the association of *Jamkesmas* on two primary outcomes of interest: health facility delivery (HFD) and skilled birth delivery (SBD). These primary outcomes were selected as they represent two key interventions that help reduce maternal mortality. Studies from LMIC show the strong correlation between maternal mortality and both HFD (*r* = −0.69 [*p* = .008]) and SBD (*r* = −0.65 [*p* = 0.006])—a﻿n increase in the proportion of women with HFD or SBD resulted in a decrease in MMR [15, 24].

In alignment with the *Jamkesmas* eligibility criteria, women in the first and second wealth quintiles were categorized as poor while women in the third, fourth, and fifth wealth quintiles were categorized as non-poor. The IDHS 2012 dataset contained a pre-calculated wealth index based on household ownership of selected assets [25]. Only women who had a live birth 5 years preceding the IDHS 2012 and were categorized as poor (first and second wealth quintiles) were selected for inclusion into the quantitative analysis. Since health insurance ownership is not a random process and is dependent on individual characteristics, we used Propensity Score Matching (PSM) to mitigate self-selection bias by balancing observed covariates between groups of poor women with and without *Jamkesmas*. PSM was used to create a new 1:1 exposed-unexposed dataset comparing poor women with *Jamkesmas* health insurance (exposed) with poor women without health insurance (unexposed) using Greedy matching technique [21] on all available socio-demographic variables: women’s age, marital status, education level, wealth, residence, employment status, sex of household head, household number, media exposure (newspaper, radio, and television), type of residence (urban versus rural), and provincial *Jamkesmas* coverage.^1^ After the creation of the new PSM dataset (*n* = 10,472), multivariable logistic regression analysis was performed to measure the association of *Jamkesmas* health insurance on HFD and SBD among poor women while controlling for all available covariates. SAS version 9.4 [26] was used for all quantitative analyses.

Qualitative methods were used to help interpret and contextualize the quantitative findings. A total of 51 in-depth interviews (IDI) were conducted with poor women who had a live birth between 2010 and 2012 (*n* = 20), midwives (*n* = 12), and government representatives (*n* = 19). The sample size for the qualitative interviews was based on recent qualitative maternal health studies conducted in Indonesia [2, 4]. Interviews were conducted from May to August 2015 on the island of Java in the province of Jakarta (*n* = 27), an urban setting with high rates of HFD and SBD, and the province of Banten (*n* = 24), a rural setting with low rates of HFD and SBD. Purposive sampling was used to recruit IDI participants. For interviews with poor women, participants were recruited in collaboration with community leaders to ensure diversity of age and health insurance experience. Midwives were recruited from the community, either from community health centers or private clinics. At the district, provincial, and ministry levels, government representatives in the maternal health and the health insurance unit (with experience in *Jamkesmas* and other national health insurance programs) were invited to participate in the interviews. Interviews were conducted in private locations and recorded to facilitate audio transcription. Electronic transcripts were uploaded into Nvivo version 10 [19] to assist with thematic framework analysis [11, 22] of qualitative data.

Ethical review and approval of this study was obtained from the Boston University Medical Center institutional review board (protocol # H-33905). Informed consent to participate in the study was obtained for participants recruited for the qualitative interviews.

## Results

### Association of health facility and skilled birth deliveries among poor women with *Jamkesmas*

The 2012 Indonesian Demographic Health Survey (IDHS) included responses from 45,607 women of reproductive age (15–49 years old). Background characteristics for the complete IDHS 2012 dataset are presented in the Appendix. Among all Indonesian women of reproductive age, close to two-thirds (63.0%) had no health insurance coverage. About one-fifth (19.1%) of all Indonesian women had *Jamkesmas* health insurance; a similar proportion (17.9%) had other forms of health insurance schemes.

Only women in the first and second wealth quintiles were included in the PSM analysis to represent the population of poor women targeted by *Jamkesmas*. In addition, the primary outcomes of interest (HFD and SBD) require women who had a live birth 5 years preceding the IDHS 2012. The background characteristics of this subset of data, before and after PSM, are presented in Table 1. Among poor women who had a live birth 5 years preceding the IDHS 2012, 72.0% of women were married, 48.1% had at least some secondary education, 47.1% did not work in the last year, 59.7% lived in households with 5 or more members, 75.0% lived in rural residence, and 68.2% lived in provinces with high provincial *Jamkesmas* coverage (Table 1).

**Table 1:** Background characteristics of poor women who had a live birth 5 years preceding the IDHS 2012, before and after propensity score matching (PSM)

	Before PSM		After PSM	
	None (*N* = 12,054)	*Jamkesmas* (*N* = 5265)	None (*N* = 5236)	*Jamkesmas* (*N* = 5236)
Age Group				
15–19	17.0%	16.1%	14.6%	16.1%
20–34	47.9%	41.8%	44.8%	41.8%
35–49	35.2%	42.2%	40.6%	42.1%
Marital Status				
Never Married	20.5%	19.9%	18.7%	19.9%
Married	73.4%	72.0%	74.8%	72.0%
Other	6.2%	8.2%	6.6%	8.1%
Education Level				
None	7.0%	5.8%	6.4%	5.7%
Completed or some primary	41.3%	46.2%	45.9%	46.2%
Completed or some secondary	46.1%	44.5%	43.6%	44.6%
Higher	5.5%	3.5%	4.1%	3.5%
Wealth Quintile				
Lowest	54.2%	59.2%	59.8%	59.1%
Second	45.9%	40.8%	40.2%	40.9%
Employment Status in Last 12 Months				
Currently employed	39.2%	37.5%	37.3%	37.6%
Not currently employed, but worked in last 12 months	17.9%	15.2%	16.6%	15.3%
Did not work in last 12 months	42.9%	47.2%	46.1%	47.1%
Sex of HH head				
Male	89.0%	87.7%	89.4%	87.7%
Female	11.0%	12.4%	10.6%	12.3%
Household Number				
1–4 members	47.1%	40.2%	40.5%	40.3%
5 or more members	52.9%	59.8%	59.5%	59.7%
Exposure to Newpaper				
None	61.8%	62.4%	63.1%	62.4%
Less than once a week	30.9%	31.0%	30.8%	31.1%
At least once a week	7.4%	6.6%	6.1%	6.6%
Exposure to Radio				
None	55.3%	51.9%	52.6%	52.0%
Less than once a week	30.2%	31.8%	31.9%	31.8%
At least once a week	14.5%	16.3%	15.5%	16.2%
Exposure to Television				
None	9.9%	9.5%	8.4%	9.5%
Less than once a week	14.8%	17.6%	17.0%	17.6%
At least once a week	75.3%	72.9%	74.6%	72.9%
Residence				
Urban	25.2%	25.1%	23.9%	25.0%
Rural	74.8%	74.9%	76.1%	75.0%
Provincial *Jamkesmas* Coverage				
Low	54.2%	31.8%	31.7%	31.8%
High	45.8%	68.2%	68.3%	68.2%

Using multivariable logistic regression from the PSM dataset, we found a positive association between the exposure variable of interest, poor women with *Jamkesmas*, and HFD and SBD (Table 2). Controlling for all available covariates, poor women with *Jamkesmas* were 19% (OR = 1.19 [1.03–1.37]) more likely to deliver in a health facility compared to poor women without health insurance. Similarly, poor women with *Jamkesmas* were 17% (OR = 1.17 [1.01–1.35]) more likely to deliver with a skilled birth attendant compared to poor women without health insurance. Other covariates also showed an association with both HFD and SBD. For example, education was shown to have a positive association with the primary outcomes of interest; poor women with the highest educational attainment were much more likely to deliver their babies in a health facility (OR = 6.43 [3.57–11.58]) and deliver with skilled birth attendants (OR = 10.68 [5.37–21.26]) compared to poor women without any education. Similarly, poor women in the second wealth quintile were more likely to have HFD (OR = 1.67 [1.44–1.93]) and SBD (OR = 1.70 [1.45–1.99]) compared to poor women in the lowest wealth quintile. Several covariates showed a negative association with HFD and SBD; poor women that did not work in the last 12 months, lived in households with 5 or more family members, and lived in rural settings were less likely to have HFD and SBD compared to their equivalent counterparts.

**Table 2:** Adjusted odds ratios (ORs) and 95% confidence intervals (CIs) for determinants of health facility delivery (HFD) and skilled birth delivery (SBD)

	Outcome = HFD		Outcome = SBD	
	Adjusted ORs	95% CI	Adjusted ORs	95% CI
Exposure variable				
Health insurance ownership (ref = none)				
Jamkesmas	1.19*	1.03–1.37	1.17*	1.01–1.35
Independent variables				
Age Group (ref = 20–34)				
15–19	1.11	0.78–1.57	0.92	0.64–1.33
35–49	1.19*	1.01–1.40	1.13	0.96–1.33
Marital Status (ref = married)				
Not married	2.66	0.22–31.86	n/a	n/a
Other	1.07	0.78–1.46	0.91	0.66–1.24
Education Level (ref = none)				
Completed or some primary	1.68*	1.09–2.59	2.25***	1.59–3.17
Completed or some secondary	3.30***	2.13–5.12	5.64***	3.64–8.07
Higher	6.43***	3.57–11.58	10.68***	5.37–21.26
Wealth Quintile (ref = first/lowest wealth quintile)				
Second	1.67***	1.44–1.93	1.70***	1.45–1.99
Employment Status in Last 12 Months (ref = currently employed)				
Not currently employed, but worked in last 12 months	0.86	0.70–1.05	0.90	0.71–1.14
Did not work in last 12 months	0.78**	0.67–0.92	0.65***	0.56–0.76
Sex of HH head (ref = male)				
Female	1.08	0.83–1.39	1.01	0.77–1.31
Household Number (ref = 1–4 members)				
5 or more members	0.74***	0.64–0.86	0.76**	0.65–0.89
Exposure to Newspaper (ref = none)				
Less than once a week	0.95	0.80–1.12	1.14	0.94–1.37
At least once a week	1.03	0.73–1.47	1.22	0.81–1.84
Exposure to Radio (ref = none)				
Less than once a week	1.27**	1.07–1.51	1.05	0.88–1.26
At least once a week	1.30*	1.04–1.62	1.18	0.93–1.50
Exposure to Television (ref = none)				
Less than once a week	1.34	0.99–1.71	1.46**	1.11–1.90
At least once a week	1.31	0.99–1.71	1.89***	1.59–2.40
Residence (ref = urban)				
Rural	0.38***	0.32–0.46	0.57***	0.47–0.70
Provincial *Jamkesmas* Coverage (ref = low)				
High	1.13	0.97–1.32	0.76**	0.64–0.89

Controlling for women’s exposure to health insurance, age, marital status, education level, wealth, residence, employment status, sex of household head, household number, media exposure to newspaper, radio, and television, type of residence, and provincial Jamkesmas coverage

n/a = too few observations to report meaningful results

*p < 0.05; **p < 0.01; ***p < 0.001

### Perceived barriers to health insurance membership and maternal health services

Results from the IDIs found key barriers associated with health insurance access and maternal health services among poor women (Table 3). Individual-level barriers to health insurance coverage included the perception that health insurance is unimportant and the difficulties in obtaining the appropriate government documents for health insurance registration. At the programmatic-level, methods of identifying *Jamkesmas* beneficiaries are not standardized and/or poorly implemented which resulted in mis-targeting of the poor.

**Table 3:** Selected statements that exemplify key barriers to health insurance access and maternal health services among poor women

Topic: Health insurance access		
*Barrier*	*Themes*	* Illustrative quotes*
Individual	Perception that health insurance is unimportant	“I’m not sick yet, so I don’t need it.” - Poor woman, Jakarta
	Lack of valid government identification	“*Puskesmas* [community health center] has already offered health insurance, but I didn’t have the documents… I didn’t have time to take care of the *Kartu Keluarga* [government family card].” - Poor woman, Banten
Programmatic	Miss-targeting of the poor	“For *Jamkesmas* there needs to be a household survey [to identify the poor], after that, the government provides health insurance for those that are poor. However, there is some nepotism that takes place so that some well-off families will also get *Jamkesmas* health insurance.” - Midwife, Banten
Topic: Maternal health service		
*Barrier*	*Themes*	*Select quotes*
Socio-cultural	Preference to deliver at parental village	“My parents are there, [if I deliver here] no one will help take care of me.” - Poor woman, Jakarta
	Use of traditional birth attendants (TBA)	“Women like to use *paraji* [TBA] because they accept whatever you have… they also help raise the baby, take care of the mother, and help with other household chores.” - Midwife, Banten
	Fatalistic point of view	“There was one woman who was delivering her fourth baby with a *dukun* [TBA]… she had heavy bleeding but did not want any assistance from a midwife or any skilled birth attendant because she believed that life and death is God’s will…both mother and baby died.” - Midwife, Banten
Accessibility	Distance to health facility	“The *puskesmas* is very far… I delivered with my midwife and *dukun* [TBA] at home.” - Poor woman, Banten
	Poor referral system	“Our referral system is a mess… there is a lot of hospital “touring” as we look for hospitals that can deal with emergency situation… as a result, we have a lot of deaths in transit.” - Government representative, Banten
	Non-facility based expenditure	“When we refer patients to higher level health facilities, they sometimes refuse. We tell them that it’s free, but they respond, “It may be free for me, but how do we pay for food for the people that will be waiting with me?”“ - Midwife, Banten
Quality of care	Shortage of qualified health providers	“I was afraid last time I was [in the *puskesmas*]… I was yelling “help doctor, help midwife, my baby is coming!” No one was there, everyone was on vacation… there was only one nurse in the *puskesmas*.” - Poor woman, Jakarta
	Overcrowded health facilities	“Here in this *puskesmas*, the number of patients is very high…the one that I met on Wednesday originally came down on Monday…ha-ha-ha… there are not enough seats in the waiting room for all the patients.” - Midwife, Banten
	Lack of health facility accreditation	“The cost associated with health facility accreditation is very high… you need to hire a consultant and a team to identify the issues… then you need a lot of resources to fix all the issues so you can be accredited… most *puskesmas* don’t have the money to be accredited.” - Government representative, Banten

Analysis of qualitative data revealed key maternal health barriers associated with socio-cultural, accessibility, and quality of care factors. Socio-cultural barriers included a preference to deliver at the parental village (where physical access to maternal health services is limited), a preference for the use of traditional birth attendants (TBA), and fatalistic viewpoints on the part of women. Accessibility was another barrier; respondents identified problems with distance to health facility, poor referral systems for higher levels of care, and non-facility expenditures such as transportation, food, and accommodation for accompanying family members. Finally, quality of care was identified as a key barrier to maternal health care in Indonesia, with issues involving a shortage of qualified health providers, overcrowded health facilities, and lack of health facility accreditation that contribute to poor quality of care.

Differences were noted between the two provinces. Interviews from Banten highlighted the challenges associated with accessing maternal health services in this province, in particular, distance to health facility and the poor referral systems. In addition, the socio-cultural factors such as fatalism and the heavy reliance on TBAs were more pronounced in the interviews from Banten compared to Jakarta. Thematic issues such as the perception that health insurance was unimportant and the poor quality of care were commonly observed in participants from both Jakarta and Banten province.

## Discussion

### Health Insurance and the Effects on HFD and SBD

The results of this study demonstrate that the recent *Jamkesmas* health insurance program in Indonesia targeted to the poor and the near-poor is positively associated with HFD and SBD. These findings contribute to the evidence-base that health insurance ownership is positively correlated with maternal health services such as HFD and SBD. Despite the statistically significant results of this analysis, the effect size for the association between *Jamkesmas* and HFD and SBD in Indonesia is modest (OR = 1.19 and OR = 1.17, respectively) in comparison to the results generated by other studies from LMICs. For example, women who had the *Seguro Integral de Salud* health insurance in Peru were twice as likely (OR = 2.0) to have HFD compared to women without health insurance [13]. In Rwanda, women who had *Mutuelles de Sante* health insurance were 60% more likely (OR = 1.6) to have HFD and more than twice as likely (OR = 2.3) to have SBD compared to women without health insurance [12, 27]. These findings support the economic theory that predicts that insurance coverage reduces the cost of healthcare expenditure to the consumer, thus resulting in higher use of health care services [38].

### Contextual Factors Affecting Maternal Health

The qualitative interviews from this study provided contextual information that offers an explanation to some of the reasons behind the modest effect of *Jamkesmas* health insurance on HFD and SBD, including socio-cultural, accessibility, and quality of care factors.

#### Socio-Cultural Considerations

Several key themes associated with socio-cultural consideration were identified from interviews with poor women, midwives, and key stakeholders, including the preference to deliver in the parental village, use of traditional birth attendants (TBA), and having a fatalistic point of view. These findings are similar to the results found in past qualitative studies conducted in Indonesia [2, 4, 31]. In Western Java, one of the main reasons that women used TBAs was the issue of trust—being part of the community, speaking the local language, and sharing the same culture meant that TBAs have developed the trust among women in the community [31]. Also, the long-standing tradition of using community-based TBAs meant the pregnant women were often encouraged by their mothers, older sisters, and close relatives to use a TBA during the delivery of their baby [2]. Fatalism was also described as a key theme in an ethnographic study in eastern Indonesia where death was a natural risk associated with delivery—community members believed that they had little control over whether women survived their pregnancy: it was ‘God’s way’ and no-one was at fault if mother or baby had a bad outcome [4].

In order to strengthen maternal health programs in Indonesia, careful planning that takes into account socio-cultural factors is important. The continued reliance on TBAs in some communities means that TBAs still have a role in improving the maternal health situation in Indonesia—either as direct health providers, referral agents to higher levels of care, or community health promoters. In addition, health promotion strategies are important in increasing community awareness about the pregnancy-related risks and the importance of skilled birth attendants and health facility deliveries. Such health promotion strategies can be delivered through public health campaigns, TBAs, or other community health promoters.

#### Accessibility

Accessibility was another key barrier that emerged, including issues such as distance to health facility, poor referral systems, and non-facility based expenditures. Previous studies have shown that proximity to heath facilities is a major factor for pregnant women in selecting delivery services [18, 20, 30, 31]. Issues such as lack of transportation options and poor transportation infrastructure can result in increased costs of health care visits. For families in poor households with limited financial means, the problem of distance is a commonly-noted rationale for using TBAs within the community [18, 31]. This emerged as a major issue in the qualitative interviews in the present study, with several women in Banten province citing distance from the community health center as the reason they elected to deliver at home. Health insurance programs should encourage women to travel to health facilities by providing coverage for referral transport and hospital stay, especially for households with limited financial means. For example, micro health insurance programs in Nepal and Jordan have benefits that include reimbursement for health facility transportation and per diems to offset hospital-related expenditure and the cost of lost wages [6, 29].

In addition, the qualitative findings identified challenges in registering for pro-poor health insurance, which in turn can prevent access to maternal health services for poor women. The *Jamkesmas* health insurance is free for the poor; however, registering for pro-poor health insurance requires the individual to have essential government identification cards and documents, including the *Kartu Tanda Penduduk* [local identity card], *Kartu Keluarga* [government family card], or *Surat Keterangan Tidak Mampu/Miskin* [certificate of financial incapability/poor]. Communities need to better disseminate the process of signing up for pro-poor health insurance and the process of obtaining proper government documentation. Moreover, local governments also need to publicize and ensure community awareness of the individual process to develop the necessary government identification and documents required for pro-poor health insurance membership. If processing fees are required for these official documents and identification cards, waivers will need to be in place for those unable to afford those fees.

#### Quality of Care

Quality of care was also identified as a key barrier to maternal health care in Indonesia. Issues involving shortage of qualified health providers, overcrowded health facilities, and lack of health facility accreditation all contribute to poor quality of care. Statements by government representatives highlighted the very real shortage of skilled birth attendants (SBA) in Banten province. This finding is in agreement with those of the World Bank, which identified a serious human resource gap in Indonesia [35]. The unequal distribution of experienced midwives and specialists (i.e., OB/GYNs) means that rural and remote areas lack adequate numbers of health care providers to provide high-quality maternal health services [35].

In addition to not having enough SBAs, the variability in clinical competence warrants additional attention. According to the WHO, a skilled birth attendant should be proficient in “the skills needed to manage normal (uncomplicated) pregnancies, childbirth and the immediate postnatal period, and in the identification, management and referral of complications in women and newborns” [32]. SBAs in LMICs may not be fully competent in providing standard of care; in Benin, Nicaragua, Jamaica, and Rwanda, an average of 56% of SBAs correctly answered all the knowledge questions and only 48% had demonstrated the correct skills [10]. This study highlighted concerns from key informants about the competence of newly trained midwives and their ability to handle obstetric emergencies. This result is similar to the findings reported by the US National Academies of Sciences in which the quality of training that midwives received was insufficient to produce competent SBAs in Indonesia; approximately 28–52% of midwives were unable to identify the presenting part of the fetus, estimate fetal weight, actively manage the third stage of labor, measure blood pressure, and provide clean and safe delivery care [16].

Another important component of quality of care is the accreditation of health facilities to ensure that hospitals and community health centers meet the national standards of care and have the necessary staff, medical equipment, supplies, and medication in order to provide high quality care. Several key informants noted that the accreditation process has not been fully implemented and enforced in Indonesia. A recent health facility survey conducted by the MOH supports this observation. The Indonesian government requires at least four facilities in each district that can perform Basic Emergency Obstetric Care (BEmOC). Unfortunately, only 61% of districts in 2011 met the minimal number of BEmOC facilities that are required by the MOH [14]. Slightly over one-fourth (28%) of these BEmOC facilities did not operate all hours daily, and less than half (45%) met the personnel requirements. More concerning is the fact that only 12% of BEmOC facilities had the necessary medical equipment and only 3% had the required medications [14].

These findings suggest that demand-side interventions alone (e.g. health insurance schemes) are unlikely to have a major impact in reducing national maternal mortality rates. They point to the need for concrete measures that also address supply-side issues such as increasing the number and distribution of qualified SBAs and accredited health facilities. Examples of supply-side interventions in LMICs that have been shown to improve maternal health outcomes include Rwanda’s performance-based financing scheme (providing financial incentives for health facilities and service providers) [27], Peru’s maternal health initiative (conducting clinical training, investing in health infrastructure development, and ensuring availability of essential medical equipment) [13], and Mexico’s social protection system in health (increasing funds to the public health system) [28].

### Study Limitations

The strength of a mixed-methods study design allows triangulation of different types of data sources in order to facilitate the interpretation of multifaceted research questions. However, several limitations in this study are important to note.

- *Quantitative analysis*: Although the PSM analysis applied a rigorous analytic approach that reduces selection bias among this observational dataset, the analysis was cross-sectional and therefore can only demonstrate an association between the key variables of interest and not causality. In addition, there are notable differences in the background characteristics of women in this PSM dataset compared to the general population of women, limiting the generalizability of the quantitative analysis. As the PSM dataset only included women in the lowest two wealth quintiles who had a live birth 5 years preceding the IDHS 2012, women in our dataset were less likely to have at least some secondary education (PSM dataset: 48.1% vs general population: 63.6%); much more likely to be unemployed in the last year (PSM dataset: 47.1% vs general population: 5.8%); more likely to live in households with 5 or more members (PSM dataset: 59.7% vs general population: 33.6%); and more likely to live in rural settings (PSM dataset: 75.0% vs general population: 47.8%). Furthermore, the exposure variable (i.e., *Jamkesmas* membership) was measured in 2012 whereas the primary outcome of interest (i.e., HFD and SBD) was collected from any live births in the preceding 5 years (any time between 2008–2012). The quantitative analysis assumes that if a woman had health insurance in 2012, she also had health insurance during the live birth in the preceding years. For example, a poor woman with *Jamkesmas* health insurance in 2012 who delivered her baby in 2010 without health insurance, would be treated in this analysis as delivering with health insurance (because she had health insurance in 2012). The opposite scenario is also possible—a pregnant woman may not have *Jamkesmas* in 2012 but had *Jamkesmas* membership when she delivered her baby in 2010. The issue of timing between the exposure and outcome variable could distort the true effect of health insurance on HFD and SBD. With the launch of *Jamkesmas* in 2008, more women were enrolled in *Jamkesmas* in 2012 compared to the early years of *Jamkesmas* implementation. Therefore, women are much more likely to have delivered their baby without health insurance during the 5 years preceding the 2012 IDHS, resulting in a potential under-estimate of current results. Finally, it is important to note that other social protection programs targeted to the poor and near poor households have been implemented during the *Jamkesmas* timeline, which may confound the true effect of *Jamkesmas*. For example, the *Jampersal* program that was implemented in 2011 provided pregnant women without any health insurance free delivery at 3^rd^ class delivery wards [1]. Unfortunately, exposure to other social protection programs were not captured in the 2012 IDHS dataset, which complicated this secondary analysis of an observational dataset. Since this analysis is focused specifically on a subset of women who are poor, we have assumed that poor women—those with and without *Jamkesmas*—have equal exposure to other social protection programs that target the poor and near poor (such as *Jampersal*) and thus there is no positive or negative association between the exposure variable (*Jamkesmas* membership) and the primary outcomes of interest (health facility and skilled birth delivery). We believe that this is a reasonable assumption as we are unaware of any empirical data that suggest that there are differential rates in *Jamkesmas* membership among the poor who have access to other social protection programs.
- *Qualitative analysis*: The qualitative interviews provide a better understanding of the contextual issues associated with health insurance membership and maternal health services. However, the difference in data collection time period may result in qualitative findings (conducted in 2015) that may not truly reflect the contextual realities observed in the quantitative survey (conducted in 2012). The study attempted to address this limitation by interviewing poor women who delivered a child between 2010 and 2012 to ensure overlap with the IDHS 2012 data collection timeframe (IDHS 2012 captured live birth data from 2008–2012) while minimizing recall bias. In addition, the study recruited midwives and government representatives who had experience with the *Jamkesmas* program and familiarity with the maternal health situation at either the national or local government level. The generalizability of the qualitative data is another potential limitation of this study. As qualitative interviews were conducted in two provinces in western Java with a small number of respondents who ﻿were selected purposively, the results from the qualitative component can only be generalizable to similar settings and may have limited transferability. Different contextual factors may be observed if respondents were recruited from different parts of the country (i.e., more remote provinces in eastern Indonesia). The study tried to address this issue by interviewing Ministry of Health officials who have a better perspective of maternal health issues nationally.

## Conclusions

Results from this study contribute to the growing UHC knowledge base in LMICs and provides evidence for the positive effects of UHC schemes on access to key maternal health services. Study findings indicate that pro-poor health insurance schemes, such as *Jamkesmas*, can increase health facility delivery and skilled birth delivery. These findings support the economic theory that health insurance coverage can reduce financial barriers to care and increase service uptake. As part of the causal chain of events to reduce maternal mortality, health insurance can be a key component that encourages women to deliver their babies at health facilities or with the aid of skilled birth attendants. However, health facilities must be fully equipped and health providers sufficiently trained in order to save the life of a woman in the event of an obstetric emergency. To make meaningful reductions in maternal mortality, factors such as socio-cultural beliefs, accessibility of facilities, ease of enrollment in the insurance plan, and quality of care are important issues that need to be addressed as part of the national UHC agenda to improve maternal health services in Indonesia.

## Appendix

**Table 4:** Background characteristics of women of reproductive age (15–49) surveyed in IDHS 2012

	IDHS 2012 (*N* = 45,607)
Age Group	
15–19	15.2%
20–34	44.2%
35–49	40.6%
Marital Status	
Never Married	21.7%
Married	73.0%
Other	5.3%
Education Level	
None	3.3%
Completed or some primary	33.2%
Completed or some secondary	51.4%
Higher	12.2%
Wealth Quintile	
Lowest	17.0%
Second	19.3%
Middle	20.3%
Fourth	21.4%
Highest	22.1%
Employment Status in Last 12 Months	
Currently employed	55.4%
Not currently employed, but worked in last 12 months	38.8%
Did not work in last 12 months	5.8%
Sex of HH head	
Male	85.2%
Female	14.8%
Household Number	
1–4 members	66.4%
5 or more members	33.6%
Exposure to Mass Media	
Reads a newspaper at least once a week	13.3%
Listens to radio at least once a week	19.3%
Watches television at least once a week	85.9%
Residence	
Urban	52.2%
Rural	47.8%
Province	
Aceh	1.9%
North Sumatera	5.3%
West Sumatera	1.9%
Riau	2.3%
Jambi	1.3%
South Sumatera	3.0%
Bengkulu	0.7%
Lampung	3.2%
Bangka Belitung	0.5%
Riau Islands	0.7%
Jakarta	4.3%
West Java	18.1%
Central Java	13.7%
Yogyakarta	1.4%
East Java	16.2%
Banten	4.7%
Bali	1.7%
West Nusa Tenggara	2.2%
East Nusa Tenggara	2.0%
West Kalimantan	1.7%
Central Kalimantan	0.9%
South Kalimantan	1.6%
East Kalimantan	1.5%
North Sulawesi	0.9%
Central Sulawesi	1.1%
South Sulawesi	3.4%
Southeast Sulawesi	0.8%
Gorontalo	0.4%
West Sulawesi	0.4%
Maluku	0.6%
North Maluku	0.4%
West Papua	0.3%
Papua	1.2%
Health Insurance Ownership	
None	63.0%
*Jamkesmas*	19.1%
Other ^a^	17.9%

^a^ Includes: Civil servant health insurance, military/veteran health insurance, social health insurance for private sector workers, private health insurance, other health insurance schemes

## Abbreviations

Askeskin: Asuransi Kesehatan Masyarakat Miskin
BEmOC: Basic emergency obstetric care
HFD: Health facility delivery
IDHS: Indonesian Demographic Health Survey
Jamkesmas: Jaminan Kesehatan Masyarakat
LMIC: Low middle income countries
MDG: Millennium development goal
MMR: Maternal mortality rate
PSM: Propensity score matching
SBA: Skilled birth attendant
SBD: Skilled birth delivery
TBA: Traditional birth attendants
THE: Total health expenditures
UHC: Universal Health Coverage

## Footnotes

1 Based on the median provincial Jamkesmas coverage rate, each of the 33 provinces was assigned a “low” (below the median) or “high” (above the median) provincial Jamkesmas coverage status.
